# CompPhy: a web-based collaborative platform for comparing phylogenies

**DOI:** 10.1186/s12862-014-0253-5

**Published:** 2014-12-14

**Authors:** Nicolas Fiorini, Vincent Lefort, François Chevenet, Vincent Berry, Anne-Muriel Arigon Chifolleau

**Affiliations:** Institut de Biologie Computationnelle, LIRMM, Université de Montpellier II - CNRS, Montpellier, France; MIVEGEC, CNRS 5290, IRD 224, Universités Montpellier 1 et 2, Montpellier, France; LGI2P, Research Centre, École des Mines d’Alès, Nîmes, France

**Keywords:** Evolutionary trees, Tree comparison, Online resource, Real-time collaboration

## Abstract

**Background:**

Collaborative tools are of great help in conducting projects involving distant workers. Recent web technologies have helped to build such tools for jointly editing office documents and scientific data, yet none are available for handling phylogenies. Though a large number of studies and projects in evolutionary biology and systematics involve collaborations between scientists of different institutes, current tree comparison visualization software and websites are directed toward single-user access. Moreover, tree comparison functionalities are dispersed between different software that mainly focus on high level single tree visualization but to the detriment of basic tree comparison features.

**Results:**

The web platform presented here, named *CompPhy*, intends to fill this gap by allowing collaborative work on phylogenies and by gathering simple advanced tools dedicated to tree comparison. It offers functionalities for tree edition, tree comparison, supertree inference and data management in a collaborative environment. The latter aspect is a specific feature of the platform, allowing people located in different places to work together at the same time on a common project. *CompPhy* thus proposes shared tree visualization, both synchronous and asynchronous tree manipulation, data exchange/storage, as well as facilities to keep track of the progress of analyses in working sessions. Specific advanced comparison tools are also available, such as consensus and supertree inference, or automated branch swaps of compared trees. As projects can be readily created and shared, *CompPhy* is also a tool that can be used easily to interact with students in a educational setting, either in the classroom or for assignments.

**Conclusions:**

CompPhy is the first web platform devoted to the comparison of phylogenetic trees allowing real-time distant collaboration on a phylogenetic/phylogenomic project. This application can be accessed freely with a recent browser at the following page of the ATGC bioinformatics platform: http://www.atgc-montpellier.fr/compphy/.

## Background

Collaborative systems are emerging as a kind of tool to help people located in different places conduct joint work. Basically, they allow several persons to edit and analyze common documents in a coordinated way. Evolutionary biologists and systematicians need to communicate, share data and interact despite being separated by long distances. This applies regardless of the extent and type of joint effort involved in, for instance, studying a few genes in a metabolic pathway, or determining the phylogeny of a specific taxonomic group based on previously published studies. Over the past few years, several collaborative tools have been proposed, such as *Teamlab* focused on project management, *Mendeley* on research papers, *GitHub* on software source code, *ShareLaTeX* on LaTeX documents and *Google Drive* on office documents. Unfortunately, none of these tools applies when *phylogenies* are the documents to be shared and jointly edited. Recently, a collaborative tool devoted to tree-building, *Phylografter* [1], was developed. It aims to facilitate the process of collaborative tree-assembly while providing links to published phylogenetic hypotheses and represents a web-based content management system for phylogenetic information that provides node-by-node provenance of grafted trees. However, in this software, the collaborative aspect corresponds to the sharing of data (trees and annotations) and tree manipulation (edition, visualization,...) is made in an asynchronous way. The bioinformatic resource proposed here, named *CompPhy*, is a good step forward. In a few words, *CompPhy* is a web platform devoted to collaborative work on phylogenies, providing facilities to jointly visualize, manipulate and mainly compare trees. Note that it allows a user to actually see on the screen changes performed by distant collaborators. This is very useful to support a phone or video conversation about a collection of trees tp overcome the need for repeated exchanges of tree files on a shared folder together with round trips with the users’ separate – hence unsynchronized – tree visualization software.

Visualizing trees is a recurrent activity in evolutionary biology and systematics due to the central role of trees in these fields. This has led to the development of more than a dozen standalone software programs to visualize trees, such as *TreeView* [2], *TreeDyn* [3], *Dendroscope* [4], *Archaeopteryx* [5], *FigTree* [6] and most recently some web tools, such as *ScripTree* [7], *iTOL* [8], *jsPhyloSVG* [9] and *EvolView* [10]. Some of these tools also allow for tree annotation. This process consists of highlighting parts of trees (tips, nodes, branches, subtrees) or whole trees with various kinds of information (*e.g.*, geographical and molecular data, references). Depending on the tool, complex annotations, such as histograms or pie charts can also be associated with subtrees to indicate the proportion of their taxa having specific properties. These are powerful tools to visualize trees individually but most of them lack features to compare trees.

Tree visualization in systematic and evolutionary biology is often done with tree comparison in mind, *e.g.*, how does the inferred tree change when tuning this or that parameter in the inference? What is the difference between trees obtained by this or that method, or from this or that dataset? How much does a gene tree differ from the species tree? Starting from this observation, *CompPhy* is built with an approach symmetrical to that of the above-mentioned software: yes, it offers tree visualization features, but with tree comparison as a main focus; its name stems from “*Comparing Phylogenies*”. Table 1 details the functionalities provided in different software and web systems with respect to the focus of the present paper. *CompPhy* is currently the only available service to compute and visualize consensus and supertrees in a browser (*i.e.*, not requiring a software installation). It is also the only web-based resource enabling the collaborative edition of a tree collection during a joint work session.

**Table 1:** **Tree comparison facilities of phylogeny visualizing tools**

**Tool**	**Year**	**Type**	**Annotations**	**Joint edit.**	**Cons./SuperT.**	***Collab.***	**Reference**
TreeView	2001	S.a.	No	No	No	No	[2]
TreeMap3	2002	S.a.	Int.	Yes	No	No	[11]
TreeDyn	2007	S.a.	Int. & scr.	Yes	No	No	[3]
EPOS	2008	S.a.	Int. & scr.	Yes	C. & S.	No	[12]
PhyloWidget	2008	Online	No	No	No	No	[13]
Archaeopteryx	2009	S.a. & Online	Int. & Scr.	No	No	No	[5]
PhyloExplorer	2009	online	No	Yes	No	No	[14]
ScripTree	2010	S.a. & Online	Scr.	Yes	No	No	[7]
TreeVector	2010	Online	No	No	No	No	[15]
iTOL	2011	Online	Scr.	No	No	No	[8]
ETE	2011	S.a. & Online	Int. & scr.	No	No	No	[16]
Dendroscope	2012	S.a.	Int. & scr.	Yes	C. & S.	No	[4]
FigTree	2012	S.a.	Int.	No	No	No	[6]
jsPhyloSVG	2012	Online	Scr.	No	No	No	[9]
Evolview	2012	Online	Scr.	No	No	No	[10]
CompPhy	2014	Online	Int. & scr.	Yes	C. & S.	Yes	This paper

*Year*: date of the last release; *Type*: some tools run as stand-alone (S.a.) software after a download/installation procedure, others can be run online, inside a web browser; *Annotations* indicates which tools allow users to annotate trees with extra information (other than just branch length or support): *Int.* (available from the interface), *Scr.* (available through a script); *Joint edit.* indicates which tools offer edit functions that jointly apply to a set of trees (compared to tools with which the same action has to repeated on each tree) *Cons./SuperT.* indicates which tools offer consensus (C.) and/or supertree (S.) computation; *Collab.* indicates which tools offer functionalities allowing collaborative work.

Basically, there are two kinds of tree comparison situations: comparing two trees, or comparing a non-trivial number of trees. We detail both of these cases below.

Several situations require side by side display of two trees to visually highlight their differences or common parts. For instance, comparing a gene tree to a species tree allows recovery of evolutionary events that shaped a gene family history [17]. Similarly, a parasite tree is often compared to a host tree to study co-evolutionary patterns [11,18]. Such a pairwise comparison situation is at the core of the *CompPhy*’s interface, whose central zone allows the user to display two trees side by side or face to face depending on where they prefer the taxa names to appear. Probably the simplest way to measure the topological difference between two trees is by computing a global distance measure that separates the trees [19]. This helps for clustering or ranking trees in a set. However, this does not pinpoint conflicting zones in the topologies of the two trees. Consensus methods are a useful work-around, displaying a summary of the topological signal common to the compared trees. Among tree visualization tools, *Dendroscope* and *Mega* [20] can compute consensus trees. But, *Mega* only allows users to compute a consensus tree in the context of a bootstrap analysis. *Dendroscope* can compute several kinds of consensus but it does not highlight agreeing or disagreeing parts of the initial trees, which is however often required to identify interesting parts in the source trees. In contrast, *TreeJuxtaposer* [21] enables simultaneous comparison of several trees, notably by highlighting structural differences and similarities between trees displayed side by side. Unfortunately this software does not run on current computers. We also refer readers to *DensiTree* [22], which provides a graphical summary of large collections of trees. It is however not suited to a detailed comparison of trees. Finally, for those familiar with the command line, the PAUP* [23] and *PHYLIP* [24] packages can be used to compute consensus trees.

In situations where a large number of trees need to be compared, consensus trees and supertrees are the most appropriate tool. Formally, consensus methods apply when all trees have exactly the same set of taxa, while supertree methods extend consensus methods to situations with unequal taxon sets [25]. Nowadays, trees are obtained more easily by automated pipelines from genomic resources, so studies tend to encompass more trees, that rarely have the same taxon set. Supertree methods are thus a tool of choice. What is really needed there is an integrated tool that allows both tree collection management and supertree computation. Indeed, in practice, an initial set of source trees is usually obtained from a pipeline or from a tree inference software program, after which they are usually cut and pasted into a file, then several scripts and programs (*e.g.*, [23,26,27]) are used for the supertree inference, and the newick form of the obtained supertree is subsequently cut and pasted into another tool to display it. The analysis is usually repeated several times to generate other supertrees by changing parameters in the supertree computation or by selecting a slightly different subset of source trees. Overall, it is quite easy to get mislead in this juggling act involving different files and tools. Unfortunately, there is a real lack of tools for managing a collection of trees and building consensus and supertrees from selected trees: *RadCon* software [28] could once be used to perform some of the above mentioned tasks, but it does not run on modern computers. Morover, there is a shortage of web tools that offer to compute supertrees, and those available do not allow tree collection management and visualization [29].

When two or more trees containing a few dozen taxa are compared, several graphical operations are needed, such as a consistent rooting of trees, a common ordering of their tips (if the potential topological disagreement allows it), differential coloration of taxa depending on the molecular, taxonomic or geographical information. Maximum comfort is achieved when these tasks can be performed in an automated way, but this can only be achieved in a tool that manages trees collectively. This excludes a good deal of the software cited above where trees have to be opened independently from one another (usually in different windows), except when scripting facilities are provided, such as in *Dendroscope* and *ScripTree*.

This outline of tree comparison functions spread out in different tree display tools, or simply missing, and the absence of tools for collaborative exploration and management of trees prompted us to develop the *CompPhy* web-based platform. Recent advances in web technologies now allow users to have web applications as powerful as stand-alone software. Such applications can be accessed from any operating system while avoiding cumbersome installation and parameterizing steps. Moreover, centralizing data on a server overcomes the need for renewed copies and updates of data among the various devices researchers generally use.

The next sections describe *CompPhy*’s architecture and functionalities. Then, an example of a typical use of the platform shows how this tool can help distant scientists to collectively analyze trees.

## Implementation

**Figure 1:**
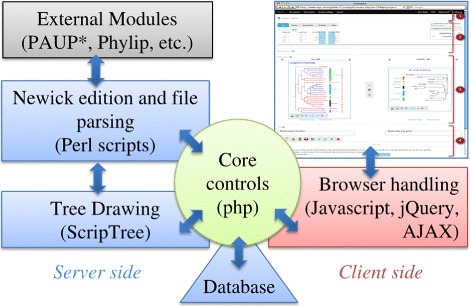
**The architecture of the** ***CompPhy*** **platform.** Projects and their trees are stored in a relational database; *ScripTree* [7] is used as the picture generating module. Perl is used for tree modifications requiring computation, *e.g.*, restricting trees to common taxa, and as a wrapper for external tree computations modules, *e.g.*, computing a consensus with PAUP* [23]. Users interact with *CompPhy* from their browser, whose content is connected to the *CompPhy* core essentially through the *jQuery* javascript framework.

*CompPhy* is based on a classic web architecture, see Figure 1. The main part is composed of PHP modules coupled to a MySQL database. The database stores information on projects and data related to trees, such as their Newick format, annotations and commands to draw them.

The *ScripTree* interpreter for phylogenetic graphics is in charge of generating tree pictures [7]. It manages tree edition and annotation. *ScripTree* takes text files as input to produce a tree picture: the tree in Newick format, a script for the graphical rendering and annotations in CSV format. However, users need no knowledge of *ScripTree* to operate *CompPhy*: they upload trees in Newick format and *CompPhy* maintains the script and annotation files according to the users’ requests on its interface. Nonetheless, a dedicated zone for the MANUAL TUNING OF THE PICTURES allows users with knowledge of *ScripTree* to tweak these files for a more specific or elaborated tree rendering. Tree pictures are generated by *ScripTree* in SVG format, which allows users to interact with pictures, *e.g.*, by pointing at taxa to be swapped, and it is now accepted by all recent browsers.

A set of Perl scripts also participates in the core of *CompPhy*: they modify the Newick format encoding trees when needed, *e.g.*, to restrict them to a subset of taxa. These scripts also interface external programs such as PAUP*, *PHYLIP*, *PhySIC_IST* and *Spruce*, in which case format transformation is often needed. Moreover, as *CompPhy* is developed according to established programming frameworks, it is relatively easy to add new computational modules in the future.

The communication between the server-side of *CompPhy* and client browsers heavily relies on the AJAX framework, which basically makes *CompPhy* behave similarly to a stand-alone application in many respects, often avoiding the need to reload the whole web page in response to users’ interactions. By adding different plugins to *jQuery*, *CompPhy* also handles the display, manipulation and refresh of SVG pictures on the client side, and this generally makes *CompPhy* more user friendly by displaying tooltips, modal boxes, etc.

*CompPhy* is hosted by the *ATGC* platform, which is managed by the *French Bioinformatics Institute* and coordinates the bioinformatics services for the South of France. Its servers run ≈190,000 hours each year for ≈11,000 different users from many countries.

## Results

**Figure 2:**
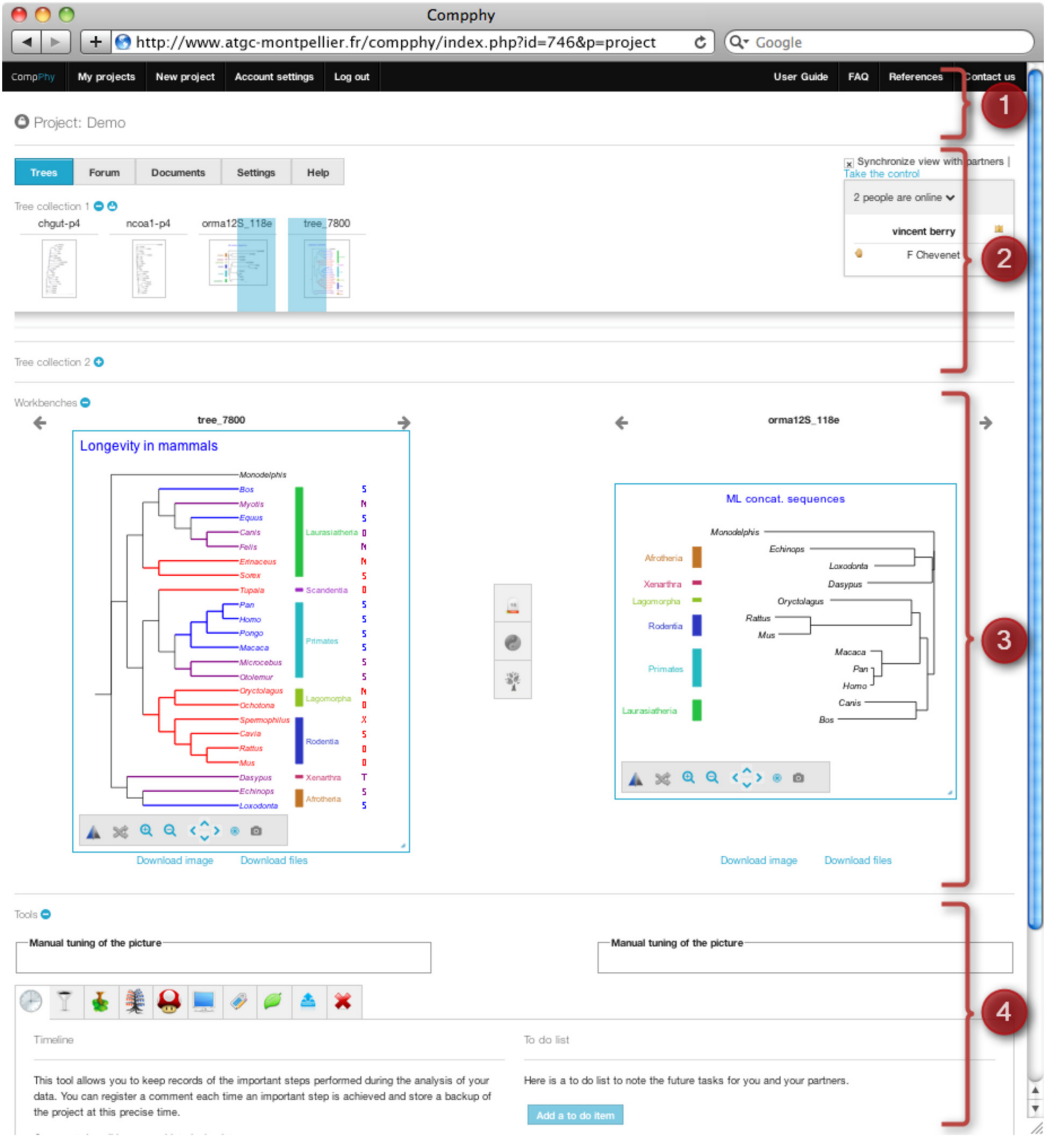
***CompPhy*** **’s main interface.** Zone 1 contains the website navigation bar. Zone 2 displays the project menu, a toolbox allowing participants to coordinate when editing trees together, and the tree collections, each tree being identified by a name and a sketched picture. Zone 3 displays two workbenches on which trees can be laid down for individual observation or comparison. Zone 4 presents several tools to edit trees and manage the project.

Trees are the central object handled by *CompPhy*. They are contained in tree collections, investigated in *projects*, each of which can be accessed by a declared list of users (possibly by anyone knowing the URL if the project is declared to be public). When working on a project, *CompPhy*’s interface can be divided in four main parts, see Figure 2.

Zone 1 contains the site menu, enabling users to navigate between their projects, to edit their account details or to access the onsite manual. Zone 2 first displays the project menu and the collaborative box (on the right-hand side) enabling users to coordinate their actions when jointly visualizing a project. For instance, this box allows them to indicate which person is in charge of a current edit. Below, it displays short captions of the project trees, organized in two collections (*e.g.*, to separate gene from species trees, or host from parasite trees). Trees can be reordered within each collection and dragged to Zone 3 to be displayed in full size. Zone 3 consists of two workbenches allowing users to display two trees side-by-side when focusing on their comparison. Operations can be performed on each tree individually or on both trees jointly: (i) tools on a workbench allow to investigate the tree it contains by zooming, resizing, flipping or translating its image, or by swapping chosen subtrees (other tools are available in Zone 4, see the paragraph below on tree edition); (ii) Zone 3 also provides pairwise comparison tools that consider the two trees displayed together on the workbenches: coordinated swap of their tips, computation of their topological distance or highlighting of their topological agreement and disagreement. At the bottom of the interface, Zone 4 contains tools that may apply to more than two trees, and tools to manage other data associated with the project (see specific paragraphs below).

Our system limits impose that you upload no more than 10,000 trees covering at most 5,000 different taxa inside a same project. Please note that we also limit the number of trees during an import to 1,000 per collection. However, CompPhy’s main focus is on collections of several dozen to a few hundred trees. Above this limit, you might not find it too convenient to use. CompPhy can easily handle trees containing more than 1,000 taxa. Above this limit you can still use it, but be aware that pictures will take longer times to load and to be rendered by your browser (they are in SVG format, which requires some computation time from the browser).

### Collaborative work

*CompPhy* allows a group of users to jointly work on a project. This work can usually be done in a number of working sessions to which a variable number of persons will participate. *CompPhy* thus proposes synchronization tools for multi-user sessions but also asynchronous tools for communication between users present and absent for some sessions. For instance, a FORUM is associated with each project, where project members can exchange questions, agree on an analysis protocol or simply leave a summary of the member’s recent work for other members who were absent at the last working session.

To coordinate users during a joint working session, *CompPhy* ensures that at any moment only one of them performs project changes. All members connected to the project are offered a synchronized (shared) view of the trees and tools. The view refreshes itself regularly, reflecting the edits done by the person in control. Insisting that only one person is in control at any moment avoids concurrent edits and ensures that a project stays in a coherent state. A COLLABORATIVE BOX (right-hand part of Zone 2 in Figure 2) indicates which project members are currently online, who is currently in control of the interface, and allows other members to REQUEST THE CONTROL in turn. Each request can be accepted or declined by either the control holder or the administrator. The latter can also TAKE THE CONTROL over the project at any moment. An option also enables a user to detach their browser from the activity performed by the others (SYNCHRONIZE tool). In this case, they can change the trees displayed on the workbenches but they can not make any concrete changes in the project, as this would interfere with the actions of the user in control.

### Data management

Data in *CompPhy* is organized around the *project* concept, that basically pools a set of analyzed trees and associated documents. Each project has an *administrator* who can invite other people to become *members* of the project. By default, projects are created with a private status, so only the project members can access the data after being identified by *CompPhy*. This policy guarantees data privacy while still allowing data sharing. In contrast, a public project can be accessed by any guest to which the URL is sent. Without opening an account on *CompPhy*, a guest can see the project trees and examine them on the workbenches. However, no guest can make changes to the trees or data of the project.

A TODO LIST reminds project members of the next tasks to be performed in the dataset analysis. Once performed, each task can be registered as an historical point in the project TIMELINE, thus keeping track of the main analysis steps. BACKUPS of the data can be built at these intermediate moments and later restored if needed.

Additional trees can be added to a project with an UPLOAD facility. Trees of the project along with their images can be downloaded one by one from the workbenches, or by collection. Extra files related to the project can also be shared between members (DOCUMENTS section), *e.g.*, documents explaining how the trees were obtained or papers related to study. It usually helps to have all information relative to a project available in one place. Tree pictures with sufficient resolution for publication can be downloaded, as well as trees and other data stored in the project in case a user wants to work offline.

### Tree edition tools

Various tree edition facilities ease the tree comparison process. As in most tree visualization programs, it is possible to color taxa or whole subtrees (COLORIZE TREES) but, importantly, this can be done here in a coordinated way for several trees together. It is possible to alter the interleaf scale and font size (DISPLAY OPTIONS) with which trees are displayed. Once again, this can be applied to a set of selected trees. Taxa names at the tips of the trees can be changed in one or several trees, and individual trees can be renamed at any time. Zoom in and out are available for trees on the workbenches to facilitate side by side comparison of trees containing different numbers of taxa.

The tree structure itself can be changed in an automated way via several operations. First, trees can be rerooted by defining a new outgroup (REROOT tool [30]). This operation can be done jointly for several trees, with several outgroup taxa being indicated in case the precise outgroup taxa differs between trees. Several outgroup levels can be indicated: when the most exterior group has no representative in some trees, a taxon from the next level is sought, and so on. The second way to alter a tree structure is by swapping branches of trees on the workbenches. This can be done on one tree (MANUAL SWAP) by selecting representative taxa of the two branches to swap, or in an automated way on the two trees in the workbenches (AUTO SWAP [31]) so that taxa appear as much as possible in the same order in both trees. Another tool enables users to RESTRICT TREES to their common taxa. This can help focus on their topological disagreement, which by definition can only derive from shared taxa arranged in different ways. In particular, gene trees obtained by phyloinformatic pipelines often have different sets of taxa, simply because genes can be lost in some species over the course of evolution.

### Distance, consensus and supertree computation

A commonly used distance to measure the disagreement between two trees is that proposed by [19]. It counts the number of clades (or bipartitions) present in one tree but not in the other. *CompPhy* proposes to indicate this value for two trees on the workbenches by interfacing *PHYLIP treedist* program [24]. This distance originally applies to two trees with identical taxon sets, so when provided with trees on different taxon sets, *CompPhy* first restricts the compared trees to their common taxa, as often done in the field.

When comparing two trees with conflicting topologies, it is often useful to highlight in evidence the largest common structure they share. Users can thus drag two trees on the workbenches and ask *CompPhy* to compute their MAXIMUM AGREEMENT SUBTREE CONSENSUS. This consensus is defined as the subtree linking the largest set of taxa whose relative placement in the two trees is exactly the same (in rare cases where several of such sets exist, one is chosen at random). *CompPhy* thus first restricts the two trees to their common taxa, then it uses PAUP* [23] to compute the consensus of the two trees, and finally displays the two trees with taxa not belonging to the consensus being shaded in light grey, while taxa in the consensus are represented with their original color. Subtrees containing taxa present in just one tree are also in grey, so that the structure of the consensus tree is clearly apparent. This has a main advantage to highlight — inside each of the two compared trees — the topological part on which they agree.

The consensus feature is thus focused on the comparison of two trees. When dealing with more than two trees, supertrees offer advantages over consensus trees. For instance, supertree methods consider taxa not present in all compared trees, whereas consensus methods overlook these taxa. *CompPhy* gives access to two SUPERTREE COMPUTATION methods: *PhySIC_IST* [32] and Matrix Representation with Parsimony (*MRP*, [33,34]). When computing the supertree by the *PhySIC_IST* method with default parameters, the degree of agreement of the input trees is translated in the resolution level of the obtained supertree: basically, a supertree containing only a few taxa and/or being poorly resolved indicates low agreement among the input trees. Changing the parameters of *PhySIC_IST* or resorting to the *MRP* method gives users an idea of the majority signal in case of substantial disagreement among the input trees (though the MRP supertree can sometimes contain artifacts representing topological signal absent from the source trees [25]). The *MRP* method is implemented via the *Spruce* library [27] to create the matrix representation of a set of source trees and via PAUP* to analyze the matrix with parsimony.

## Discussion

Progress in computing technologies has considerably boosted the potential of web browsers, which are now a real alternative to stand-alone software for different tasks. Together with *cloud sourcing* facilities (data being stored online on trusted servers), they overcome the need to install specific software while still offering the possibility to access and edit data from different devices (desktop or laptop computer, smartphone, tablet), the underlying operating system as well as the place they are in (work, home, airport, visited lab). The tool presented in this paper was created under this cloud philosophy for all the reasons listed above. It is hosted on a bioinformatics platform that has been receiving for ten years requests from several hundreds to several thousands users a month.

Most of all, *CompPhy* relies on web technologies to be a *collaborative* tool. Over the course of their activity, systematic and evolutionary biologists are involved in a number of phylogenetic projects with other local or distant people, such as academics, co-workers in the industry, and students. For all of these projects, there are always cumbersome and repeated steps for exchanging the data, converting them into the format required by the tool used by each person. Moreover, for distant co-workers, there is a difficult task of explaining and discussing the changes that were done and those to be done, or simply discussing what a collection of trees highlights in one’s data, which is best done by jointly visualizing the collection.

Consider the following illustration where *CompPhy* could be highly useful. Mr S., a PhD student, works on horizontal gene transfers (HGT) among bacteria, in collaboration with Mr B. (lab. of infectious diseases) and Mrs C. (lab. of computer science). Mr S. invites his collaborators to access the project (PROJECT SETTINGS) containing his gene trees, a comprehensive tree computed by a supermatrix approach and other documents (alignments, articles, etc). After logging onto the site, Mr B. and Mrs C. can visualize, compare and edit the tree collections of the project. They first do this asynchronously: Mr B. (from a mobile device remote from his lab) colors some taxa (COLOR TREES) in order to clarify the data; then Mrs C. edits gene trees and reroots some of them (REROOT TREES) to ease the comparison. Then they each leave a message (FORUM) to briefly explain their edits. The three of them plan a common working session during which they both connect to *CompPhy* and to a video conference system. Once logged onto the project, each participant can easily know who is currently in control of the interface (COLLABORATIVE BOX). During the meeting, all participants have a synchronized view (SYNCHRONIZE TOOL) and can see the tree collection changing in real time when the one in control performs the edits. Mr S. presents his first conclusions on the presence of HGTs by highlighting on the shared visualization page the species suspected to be involved in HGTs (COLOR TREES). Mr B. requests to be in control of the interface (COLLABORATIVE BOX). After several branch swaps in the species tree (MANUAL SWAP), Mr B. advocates that part of Mr S.’s conclusions are wrong due to a species group being misplaced in the supermatrix tree with respect to current taxonomic knowledge. He identifies a gene tree responsible for this topological problem (AUTOMATIC SWAP, and MAXIMUM AGREEMENT SUBTREE CONSENSUS), corrects it, then asks Mr S. to redo the supermatrix analysis. The latter then uploads the new comprehensive tree (UPLOAD TREES and TREE NAMES). Eventually, they end up with a consistent coloring of the gene trees and of the species trees showing evidence of HGT events in a particular gene tree. To use the tree pictures in an article, they standardize the taxon names in their different trees (RENAME TAXA), they add annotations corresponding to the tips of their trees to indicate taxonomic groups (MANUAL TUNING) and make a backup of the project (BACKUP tool).

Though *CompPhy* is designed for researchers, it can also be used for educational purposes. Practical sessions with undergraduates are ready to set up as this tool requires no specific installation: students only need a web browser for the session. Before the session, the lecturer can prepare data to be visualized by the students by creating a *public* project, which grants access to students as soon as they are communicated the project URL. When a lecturer wants students to perform tree analyses, he can ask them to create accounts and projects, inviting him to their project so that he can later access online or collect their grouped or individual homework.

The fact that *CompPhy* offers numerous features for collaborative work does not impede users from creating personal projects, *i.e.*, hosting of the data (*i.e.*, granting them access to an updated data whatever the computer or device used) and the various tree comparison tools.

Several extensions of *CompPhy* are currently being investigated, including a versioning system of individual trees. This would enable users to keep track of the various versions of their trees and hence bring the “undo” facility. Other pairwise comparison and consensus methods should be available soon. We are also planning to accept more tree formats as input (*e.g.*, Nexus and PhyloXML) and to implement more real-time features such as onsite audio/video calls and chat.

## Conclusion

*CompPhy* is the first online platform allowing several users to synchronously or asynchronously handle phylogenetic trees in a collaborative way. It allows them to see the actions performed by the others in real time on compared trees, which greatly facilitates joint work from distant places. Moreover, *CompPhy* is a unique tool pooling tree comparison operations such as restriction to common taxa, automatic branch swap, consensus and supertree computation, whose results can be readily visualized in its interface. Finally, it offers an interface for usual tree edition facilities, such as leaf and subtree coloring, subtree swapping and tree rerooting.

## Availability and requirements

**Project name:***CompPhy***Project home page:**http://www.atgc-montpellier.fr/compphy/**Operating system(s):** Platform independent **Other requirements:** A recent internet browser**License:** The tool is available online free of charge, and code is available under GitHub (https://github.com/Johy/CompPhy)**Any restrictions to use by non-academics:** None
